# Gender and Timing during Ontogeny Matter: Effects of a Temporary High Temperature on Survival, Body Size and Colouration in *Harmonia axyridis*

**DOI:** 10.1371/journal.pone.0074984

**Published:** 2013-09-25

**Authors:** Michal Knapp, Oldřich Nedvěd

**Affiliations:** 1 Department of Ecology, Faculty of Environmental Sciences, Czech University of Life Sciences Prague, Prague, Czech Republic; 2 Department of Zoology, Faculty of Science, University of South Bohemia, České Budějovice, Czech Republic; 3 Department of Biochemistry and Physiology, Institute of Entomology, Biology Centre AS CR, České Budějovice, Czech Republic; UCLA, United States of America

## Abstract

The ambient temperature experienced during development is a crucial factor affecting survival and adult phenotype in ectotherms. Moreover, the exact response of individuals to different temperature regimes is frequently sex-specific. This sex-specific response can result in varying levels of sexual dimorphism according to the experienced conditions. The majority of studies have investigated the effects of temperature on individuals reared under a constant temperature regime throughout their whole preimaginal development, whereas information on stage-dependent variation in temperature effects is scarce. Here we investigate how the stage at which elevated temperature is experienced influences survival, adult body size and colouration in the harlequin ladybird *Harmonia axyridis* form *succinea*. The effects of timing of exposure to elevated temperature on the adult phenotype are assessed separately for males and females. Control individuals were reared at a constant temperature of 20°C. Beetles in other treatments were additionally exposed to 33°C for 48 hours during the following developmental stages: egg, 1^st^ to 2^nd^ larval instar, 3^rd^ larval instar, 4^th^ larval instar and pupa. Exposure to an elevated temperature during the early developmental stages resulted in lower survival, but the adult phenotype of survivors was almost unaffected. Exposure to an elevated temperature during the later developmental stages (4^th^ larval instar or pupa) resulted in the decreased melanisation of elytra, decreased structural body size and increased dry mass. Furthermore, the timing of high temperature exposure affected the degree of sexual dimorphism in elytral melanisation and dry mass. We demonstrate that the effects of elevated temperature can vary according to the developmental stage at exposure. Detailed information on how ambient temperature affects the developmental biology of ectotherms is crucial for modeling population growth and predicting the spread of invasive species such as *Harmonia axyridis*.

## Introduction

Environmental conditions play a crucial role in the development of individual organisms because many crucial life-history traits demonstrate a considerable degree of phenotypic plasticity [1], [2]. In ectotherms, ambient temperature is among the most important environmental variables influencing development and adult phenotype. Ambient temperature is so important because these organisms are almost unable to regulate their internal temperature independently of the surrounding conditions. Complex interactions between temperature and life-history traits occur because temperature affects virtually all biochemical processes.

Body size is considered to be one of the most important traits in insects. There is, for example, a strong relationship between female body size and potential fecundity [3]. Adult body size is usually negatively related to developmental temperature in ectotherms. This relationship, often referred to as the temperature-size rule, has received broad attention in the last few decades [4]–[6]. Growth rate (increase in mass per day) and developmental rate (progress in development – i.e. reciprocal of development duration in days) differ in their response to temperature. This decoupling of temperature dependence in growth rate and development rate has been identified as probable mechanism responsible for the change in body size with temperature. Such mechanism is able to account for the existence of the temperature-size rule as well as exceptions to the rule [7], [8]. However, there is only limited knowledge of how the decoupling of these rates progresses throughout ontogeny and which life stages are most sensitive to temperature changes with respect to adult body size [6].

In several insect species, including the ladybird *Harmonia axyridis* form *succinea* (investigated in the present study), the temperature experienced during immature stages determines adult colouration [9], [10]. The cuticle of individuals reared at lower temperatures contain more melanin than of those reared at higher temperatures [9]. Lower reflectance increases the rate at which body temperature can be raised during sun basking. Thus lower reflectance greatly increases adult fitness in environments with suboptimal ambient temperatures [11], [12]. Phenotypic plasticity with respect to melanisation may be especially important if the ambient temperature varies at timescales comparable to adult longevity [13], [14]. Variation at substantially shorter as well as longer temporal scales should lead to selection against phenotypic plasticity and towards stable trait value [14].

The majority of adult holometabolous insects are not able to alter their colouration (reflectance) during their lifetime (but see [15]). In species with adult colouration determined during preimaginal development, the exact period of sensitivity to environmental cues (e.g. temperature) is expected to be late in preimaginal development. This is because the environmental conditions experienced by adults are expected to be close to those experienced by the latest immature stages [10]. A previous study on *H. axyridis* indicated that the period of sensitivity may be quite long in this species and melanisation increased linearly with the length of time spent at cold temperature [13]. This contradicts the hypothesis that the effect of temperature should increase until adult colouration is established.

Temperature can also substantially affect the preimaginal survival of ectotherms [16]–[18]. Egg-to-adult survival is a composite trait determined by multiplicative effects of stage-specific survival [17]. Interestingly, the stage at which survival rate is most sensitive to temperature varies among species, ranging from egg to early and late larval instars and pupa [16], [17], [19]–[21]. To our knowledge, similar data are scarce even for well studied species such as *H. axyridis* (but see indicia reported in [22], [23]).

In many insect species there are differences between males and females in various life-history traits. Body size is the most thoroughly investigated sexual dimorphic trait in insects [24]–[26]. The extent of sexual dimorphism may vary among populations [24], [26], [27]. Intraspecific variation in sexual size dimorphism stems mainly from sex-specific selection pressure that can differ among populations. However, sex-specific differences in phenotypic plasticity are also partially responsible for such variation [27], [28]. Unfortunately, little is known about how sexual size dimorphism varies throughout ontogeny (but see [29], [30]). Even less is known about the sexual dimorphism of other traits, such as melanisation.

The recent changes in global climate do not simply mean a general increase in temperature but may cause more frequent occurrences of extreme weather events [31], [32]. For example, several severe heat waves have occurred in the northern hemisphere since the beginning of the 21^st^ century [33]. The effect of these periods of elevated temperature on ectotherms could vary with the developmental stage at which they are felt. Unfortunately, our current knowledge on stage-specific temperature effects is limited to a few species, mostly pests in storage buildings (e.g., [20], [21]).

In this study, we investigated the effect of transient periods of elevated temperature (close to the maximum physiologically suitable temperature) on the survival, adult structural body size, dry mass and elytral melanisation of the ladybird beetle, *Harmonia axyridis*. This effect was tested on a range of developmental stages. Moreover, reaction norms are reported separately for males and females. This enabled us to investigate emergence of sexual dimorphism in body size, dry mass and melanisation of adults during the course of preimaginal development.

## Materials and Methods

### The Study Species and its Temperature Requirements

**Figure 1:**
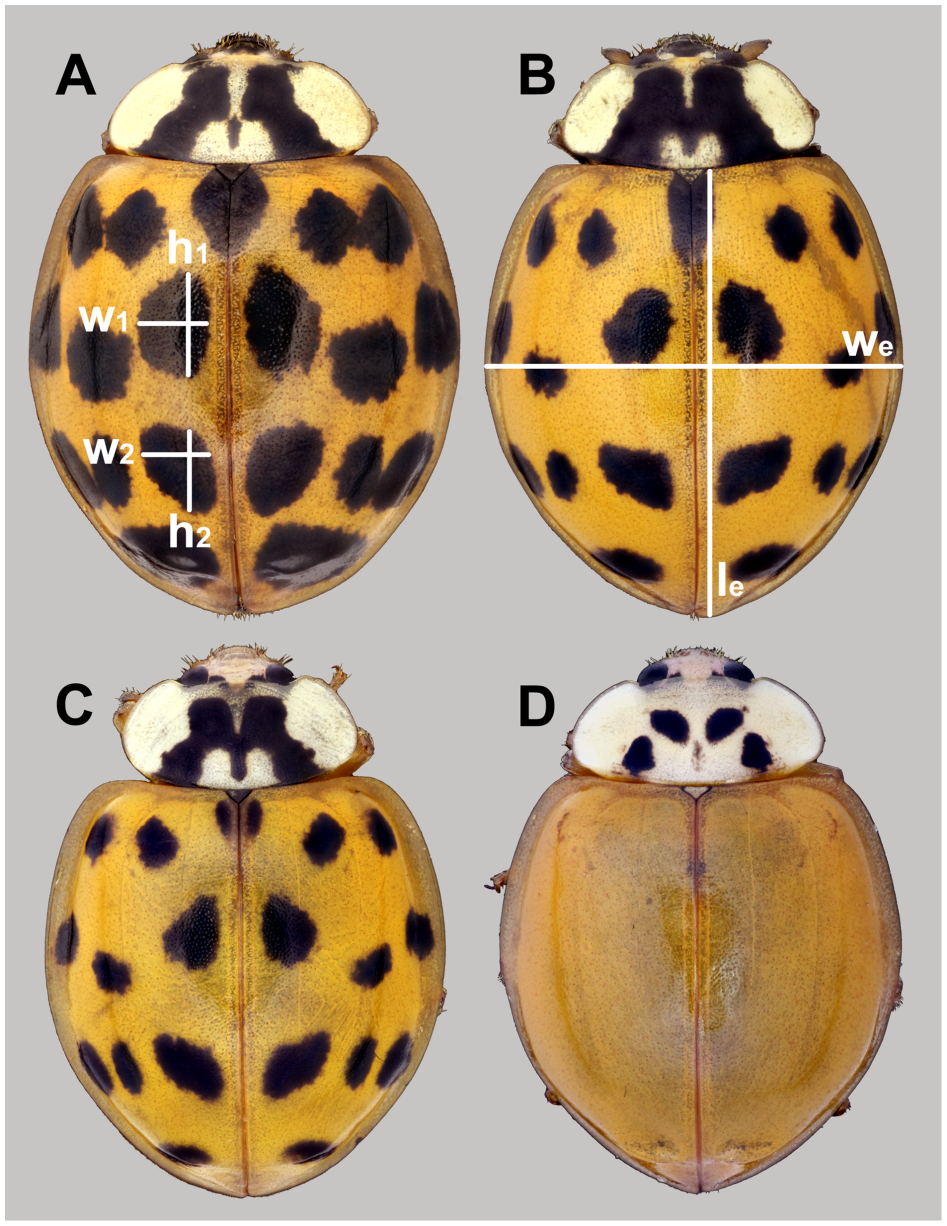
An illustration of female and male phenotypes resulting from various temperature treatments. Particular panels display: A) female reared at constant temperature 20°C; B) female reared at constant temperature 20°C and exposed to 33°C for 48 hours during pupal stage; C) male reared at constant temperature 20°C; D) male reared at constant temperature 20°C and exposed to 33°C for 48 hours during pupal stage. White bars represent dimensions measured for each specimen: l_e_ = elytron length; w_e_ = elytron width; h_1_ = spot no. 1 height; w_1_ = spot no. 1 width; h_2_ = spot no. 2 height; w_2_ = spot no. 2 width.

*Harmonia axyridis* (Pallas, 1773) is an invasive aphidophagous ladybird species (Coleoptera: Coccinellidae) from East Asia. It has been rapidly spreading across North America and Europe during recent years [34], [35]. Alien invasive populations of this species have been established mostly in areas with a temperate climate (except hot coastal Kenya [36]). The beetle is variable in body size: 5–8 mm long, with several genetically distinct forms which differ in their colouration. In Europe, the most widespread form is *succinea* (86% in the Czech Republic; [37]), which is also investigated in the present study (Figure 1). This form is known for its thermal melanism: individuals reared at lower temperatures (about 20°C) have pronounced black spots on their elytra, whereas those reared at higher temperatures (above 28°C) are pale and almost completely lacking these spots [9]. *H. axyridis* is also characterised by extremely high lifetime fecundity (up to 3,819 eggs; [38]), with daily egg production reaching up to 86 eggs in one or two clutches [39]. The number of eggs produced is highly variable and depends on the environmental conditions, particularly the availability of food [40].

The anabolic rate (i.e. biosynthesis) of *H. axyridis* is maximized at low temperatures of around 5°C whilst the catabolic rate is maximized at 25–30°C [22], [41]. The lower developmental threshold for *H. axyridis* larvae is about 11°C (as measured on a diverse range of food types and in several populations) [23], [42], [43]. Temperatures between 14 and 28°C were found to be suitable for the development and reproduction of *H. axyridis* in Great Britain [9] and similar results were reported for populations introduced to Brazil [44]. The upper development threshold was estimated to be between 30°C and 35°C by Acar et al. [22]. Only 25% of first instar larvae reached adulthood at 34°C [23], and beetles suffered substantial mortality (exceeding 50%) even at constant temperature of 33°C (O. Nedvěd, unpublished data).

### Experimental Design

The parental individuals used in the present study originated from a laboratory colony. The beetles used to establish this laboratory colony were collected in 2011 from shrubs in the university campus in České Budějovice, Czech Republic (48°59′N, 14°27′E, 400 m a.s.l.). No permits were required for the described study (manipulation and laboratory rearing of *H. axyridis*). The study complied with all relevant national regulations. Landowner provided us with the permission to enter the area and collect beetles. The colony was bred in a photoperiod of 18L:6D at 20, 25 and 20°C (six hours period at each temperature) during the photophase and 15°C during the scotophase. The relative humidity was kept to 70%. Beetles were fed ad libitum with pea aphids (*Acyrthosiphon pisum* (Harris, 1776)) grown on *Vicia faba* L. Beetles of the second laboratory generation were sexed early after adult eclosion and kept separated to ensure that all females remained unmated until the start of the experiment.

Twenty males and twenty females were used to form parental couples. Each couple was placed into a separate Petri dish (9 cm in diameter) containing crumpled filter paper strips, which provide suitable substrate for egg laying. Petri dishes were kept in a climatic chamber (Sanyo® MIR-155) at constant temperature of 20°C, relative humidity of 70% and a photoperiod 16L:8D. Beetles were fed daily with pea aphids and water ad libitum was provided in 1.5 ml Eppendorf® tubes stopped with cotton-wool. Petri dishes were checked for eggs on a daily basis. Egg clutches of over 20 eggs were included in our experiment, whereas the others were discarded. The eggs of the first six clutches included per each parental couple were counted and assigned at random to one of the six experimental treatments (see Table 1). Petri dishes with eggs were subsequently placed into the same conditions as the adults, or alternatively at 33°C (treatment E).

**Table 1:** Description of the experimental treatments employed to test the effects of elevated temperature at various stages of preimaginal development in *Harmonia axyridis*.

Name	Treatment description
C	Complete preimaginal development took place at 20°C (control treatment)
E	Development took place at 20°C, but eggs were exposed at 33°C for 48 hours
L1	Development took place at 20°C, but the 1^st^ and partially the 2^nd^ larval instars were exposed at 33°C for 48 hours*
L3	Development took place at 20°C, but the 3^rd^ instar larvae were exposed at 33°C for 48 hours
L4	Development took place at 20°C, but the 4^th^ instar larvae were exposed at 33°C for 48 hours
P	Development took place at 20°C, but pupae were exposed at 33°C for 48 hours

* The first larval instar in *H. axyridis* subsists for a shorter time than 48 hours at 33°C, therefore it was necessary to make pooled treatment for the 1st and 2nd instars (see Table S1).

Egg clutches were checked every morning and when larvae hatched, food (*A. pisum*) and water (in Eppendorf® tubes stopped with cotton-wool) were added in excess. The number of hatched individuals was recorded. Food and water were renewed daily. The first and second instar larvae were reared together (complete clutch) in one Petri dish. When larvae moulted to the third instar, the number of surviving larvae was counted and 12 individuals were selected at random (if there were 12 individuals or less, all available individuals were used). These larvae were assigned randomly to three new Petri dishes (four individuals in each) to reduce encounters between these larger larvae. Surplus individuals were discarded from the experiment because of the limited amount of aphids we were able to produce. Finally, the number of surviving individuals was recorded for teneral adults. Petri dishes from a particular treatment were moved in the course of the experiment between climatic chambers set to 20°C and 33°C according to the scheme denoted in Table 1. For information on development times of particular stages at 20°C and 33°C see Table S1.

Teneral adults were kept alive for 48 hours at 20°C to ensure complete hardening and full melanisation of their exoskeleton [9]. Afterwards, adults were sexed and killed by freezing and individually stored in a freezer as recommended by Knapp [45]. All adults were photographed using a Leica® stereomicroscope EZ4D with built-in 3 megapixel digital camera. Structural body size (elytron length and width) and the dimensions of two selected melanised spots (see Figure 1) were measured from the photographs using ImageJ software, version 1.44 [46]. Beetles were then dried for 48 hours at 50°C and their dry mass was measured using a Sartorius® CP225D-0CE microbalance with a precision of 10^−5 ^g.

### Data Analysis

**Figure 2:**
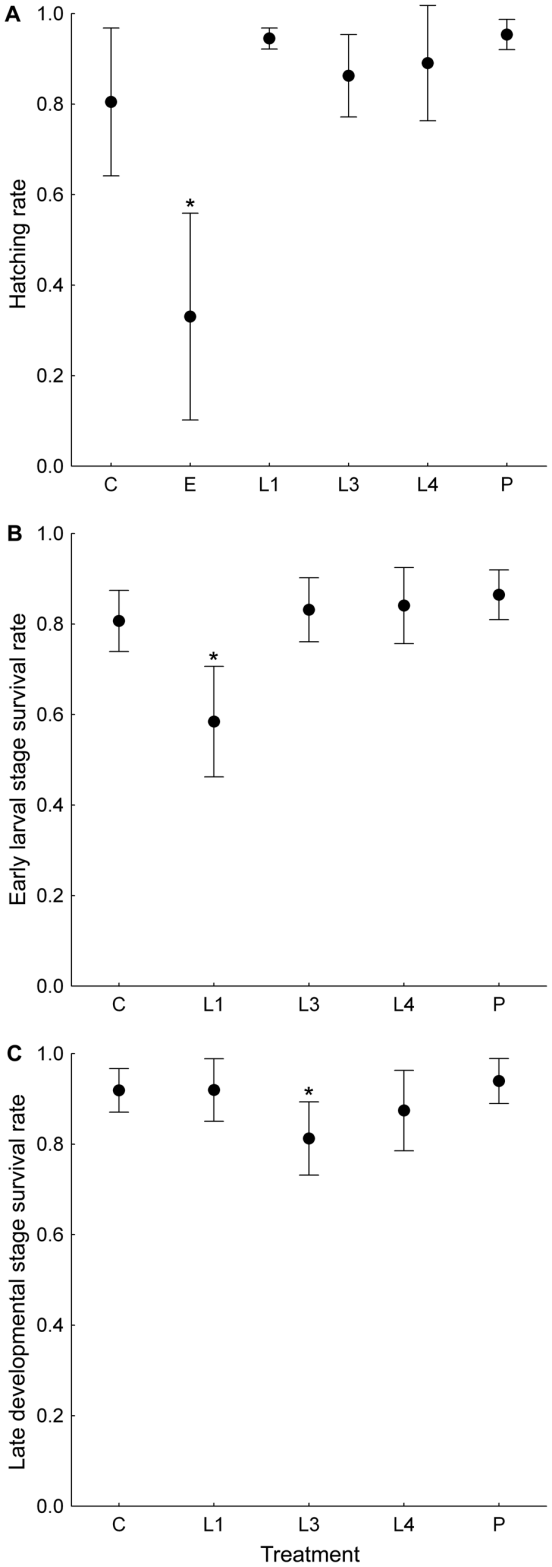
The effect of timing of exposure to elevated temperature on the survival rate of various developmental stages of *Harmonia axyridis*. Mean survival rates±1.96 SE of individuals exposed to six various temperature treatments (see below) are shown for A) eggs ( = hatching rate), B) early larval stages (1^st^ and 2^nd^ larval instars) and C) late developmental stages (3^rd^ larval instar, 4^th^ larval instar and pupa). Particular treatments employed were: “C” - beetles reared at constant temperature (20°C); beetles reared at constant temperature (20°C) with the exception of period with elevated temperature (48 hours at 33°C) during egg stage (“E”), 1^st^ and partially 2^nd^ larval instar (“L1”), 3^rd^ larval instar (“L3”), 4^th^ larval instar (“L4”) or pupal stage (“P”). Asterisk indicates significant difference (P<0.05) in survival rate between tagged treatment and control treatment within a particular panel.

Data were analysed using generalised linear mixed models (GLMM) in R 2.14 [47]. Models were fitted by maximum likelihood assuming a Laplace approximation of the likelihood function and using a penalized iteratively re-weighted least squares algorithm, as implemented in the “glmmPQL” function of the “nlme” package [48]. In all models, the clutches affinity to the parental couple (family) was used as random factor. At the first step, the random factor (family) was used for both slope and intercept. However, the influence of the random effect on the slope was not significant in any model. Therefore models with only the intercept of the random factor are presented. Three clutches from one parental couple (family) completely failed to hatch, so we decided to discard this family from the final analyses, resulting in a total of 11 families analysed. Because of the limited number of larvae that hatched from eggs in treatment E (there were less than three live hatchlings in six families from the 11 analysed; see Figure 2A), there was a lack of data for subsequent analyses of this treatment. Therefore, we decided to present analyses (other than egg survival rate) with data omitted for treatment E.

To analyse the effect of elevated temperature on the survival rate of beetles at various developmental stages, we defined three survival rates: 1) hatching rate, i.e. the proportion of hatched first instar larvae from total number of eggs laid per particular clutch; 2) early larval survival rate, i.e. the portion of larvae that successfully moulted to the third instar from the total number of first instar larvae hatched; 3) late development phase survival rate, i.e. the portion of live teneral adults from the total number of third instar larvae maintained in the experiment (max. 12 beetles per clutch). Survival rates were analysed using GLMMs with a binomial distribution of errors.

To analyse how increased temperature influenced the body size and the degree of melanisation of adult ladybirds at different developmental stages we used GLMMs with a Gamma distribution of errors for body size and a binomial distribution of errors for melanisation rate. In these analyses we also tested for sex-specific effects, in addition to temperature treatment and sex we included the sex × temperature treatment interaction term. Separate analyses were performed for dry body mass and for structural body size (i.e., body size measurements not varying during the adulthood of a particular animal; [49]) represented in this study by approximated elytron area (A_e_). Area was estimated as elytron length multiplied by elytron width (Figure 1). The degree of melanisation represented the approximate portion of the elytron covered by two selected spots and was calculated as follows: 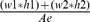. For a description of the measures used for computation of melanisation see Figure 1. All analyses of adult body size and melanisation were performed on a dataset that excluded retarded individuals. Such beetles were notably smaller, they had some deformed body parts and their juvenile development lasted much longer in comparison to other beetles from the same clutch. In total, 10 of 604 beetles were excluded from the complete dataset (including beetles from all treatments) and 545 beetles remained in dataset for final analyses (beetles from treatment E were omitted).

To identify significant differences among particular treatments in analyses of survival and among particular treatments, sexes and their combinations (interactions) in other analyses, post-hoc testing was performed. Appropriate linear contrasts were set up and tested using “glht” function as implemented in the package “multcomp” [50].

To provide at least a partial insight into the effects of elevated temperature during egg development on survival during later developmental stages, and on adult body size and melanisation rate, we compared beetles from treatment E to the control beetles. However, such analyses were based only on limited number of families. Families with no hatched individuals in treatment E were omitted from survival rate analyses, and additional families, i.e. those with adults of only a single sex, were discarded from body size and melanisation analyses.

## Results

### Survival

Exposure of eggs to 33°C for 48 hours (treatment E according Table 1) caused a significant decrease in hatching rate in comparison to eggs that were exposed to a constant temperature of 20°C (GLMM-binomial: F_5,50_ = 11.50; P<0.001; Figure 2A). Exposure of the first instar larvae (L1) to 33°C for 48 hours significantly decreased survival rate in comparison to larvae exposed to 20°C (GLMM-binomial: F_4,40_ = 10.16; P<0.001; Figure 2B). Exposure to 33°C for 48 hours also significantly decreased the survival rate of the third instar larvae (L3) when compared to the control larvae (C) (GLMM-binomial: F_4,40_ = 3.05; P = 0.028). No effect of exposure to 33°C on survival was observed for the fourth instar larvae (L4) and for pupae (P) (Figure 2C).

Based on analysis of the reduced dataset, larval survival rates of beetles exposed at 33°C during the egg stage (E) did not differ significantly from those reared at constant 20°C (P>0.15; Figure S1).

### Body Size

**Figure 3:**
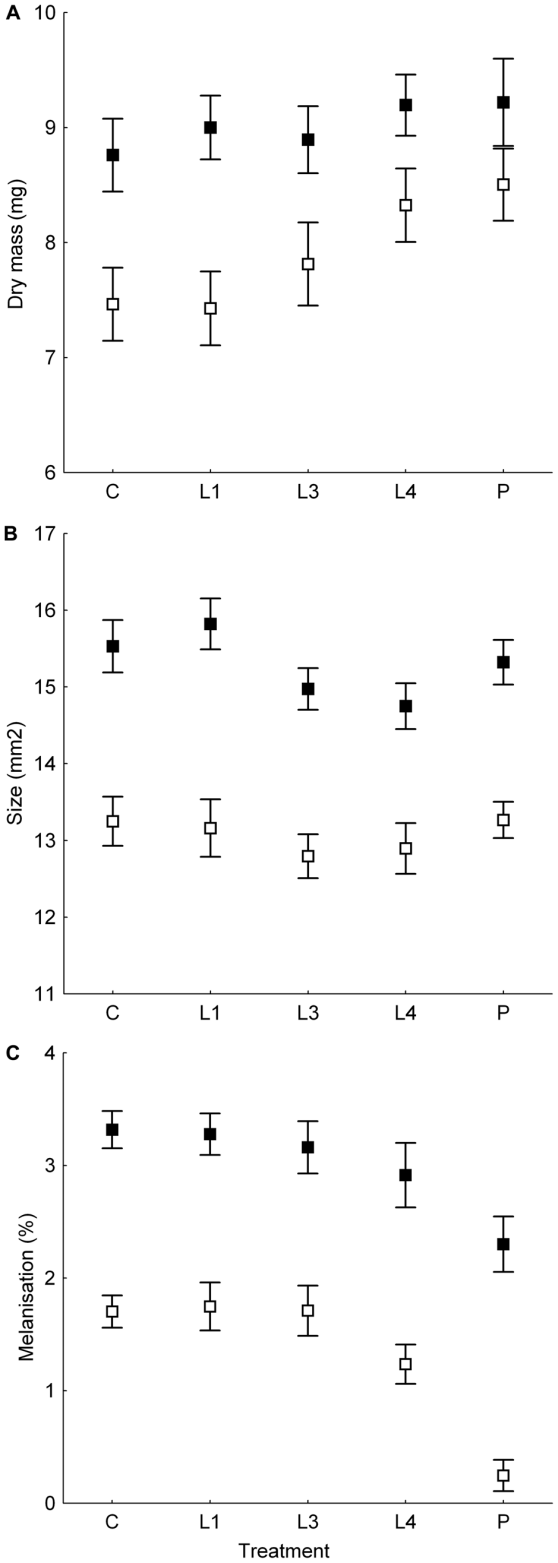
The effect of timing of exposure to elevated temperature on body size and degree of melanisation of adult *Harmonia axyridis*. Dry mass (A), structural size - approximated elytron area (B) and degree of melanisation displayed as a percentage of approximated elytron area covered by two melanised spots measured (C) of individuals exposed to various temperature treatments during preimaginal development (see below) are shown. Particular treatments employed were: “C” - beetles reared at constant temperature (20°C); beetles reared at constant temperature (20°C) with the exception of period with elevated temperature (48 hours at 33°C) during 1^st^ and partially 2^nd^ larval instar (“L1”), 3^rd^ larval instar (“L3”), 4^th^ larval instar (“L4”) or pupal stage (“P”). Means and ±1.96 SE are displayed separately for males (open squares) and females (closed squares). For detailed information on significant differences between sexes, particular treatments and interactions see Table S2.

Dry mass of adult ladybirds was significantly affected by their sex, temperature treatment and the interaction between these two variables. Females were significantly heavier than males (on the average 1.14 times). Beetles exposed to 33°C during their fourth larval instar (L4) or during their pupal stage (P) were significantly heavier than the control beetles (C) and this pattern was more obvious for males (Table 2; Figure 3A; Table S2).

**Table 2:** The effects of sex and temperature treatment on the degree of melanisation and body size in adult *Harmonia axyridis* beetles.

		Melanisation		Dry mass		Structural body size	
Term	Df	F-value	P-value	F-value	P-value	F-value	P-value
Sex	1	374.09	**<0.001**	131.49	**<0.001**	698.59	**<0.001**
Treatment	4	33.37	**<0.001**	10.64	**<0.001**	9.57	**<0.001**
Sex×Treatment	4	23.02	**<0.001**	3.10	**0.015**	1.26	0.285
Residual	525						

The results of generalised linear mixed effect models with family (parental pair) included as random factor are reported. Significant terms (P<0.05) are highlighted in bold.

Structural body size of adult beetles was significantly affected by their sex and temperature treatment. Females were larger than males and beetles which were exposed to 33°C as third or fourth instar larvae (L3, L4) were smaller than control beetles (C) (Table 2; Figure 3B; Table S2).

Based on analysis of the reduced dataset, the structural size of beetles exposed to 33°C during egg stage (E) was slightly higher in comparison to those reared at constant 20°C (P = 0.047; Figure S2A), whereas there were no differences in their dry mass (P = 0.44; Figure S2B).

### Melanisation

The degree of melanisation observed in adult ladybirds was significantly affected by their sex, temperature treatment and the interaction between these terms. Males were significantly less melanised in comparison to females. Beetles exposed to 33°C in their late developmental stages (L4, P) were less melanised than the control beetles (C) and this pattern was much more obvious for males (Table 2; Figure 3C; Table S2).

Based on analysis of the reduced dataset, there was no difference in melanisation between beetles exposed to 33°C during egg stage (E) and those reared at constant 20°C (P = 0.80; Figure S2C).

## Discussion

The influence of ambient temperature experienced during juvenile development on adult phenotype is well documented for ectotherms (e.g., [8], [9], [51]–[53]). However, detailed information on variation of temperature effects throughout the course of juvenile development of insects is scarce. Our results demonstrate that the effects of a transient period of elevated temperature are dependent on the timing of exposure. A period of elevated temperature influenced all investigated traits (dry mass, structural body size and the degree of melanisation). Moreover, the timing of the period of elevated temperature considerably affected the survival rate of *H. axyridis.*

Theoretically, the survival rates of the egg and pupal stages should be less affected by extreme temperatures than that of mobile larvae and adults that can escape from the heat through behavioural avoidance [17], [54]. However, this is often not the case in beetles. For example, lethal time at 42°C for eggs and pupae of the drugstore beetle *Stegobium paniceum* was much shorter than for larvae and adults [21]. In *H. axyridis*, we found a decrease in mortality with later timing of the period at elevated temperature. Eggs were the most sensitive to heat whilst pupae were the most resistant. Exposure to 33°C probably caused physiological stress to early juvenile stages, while pupae were able to survive exposure at much higher temperatures. In a subsequent experiment, we found that *H. axyridis* pupae were able to complete development even at 38°C (unpublished data).

The pattern of stage-dependent survival rate that we found in *H. axyridis* was also observed in the confused flour beetle, *Tribolium confusum* [20]. A possible explanation for the high sensitivity of eggs to elevated temperature could be that the amount of oxygen obtained by diffusion across the eggshell increases with temperature more slowly than the metabolic rate of the developing insect [55]. However, this mechanism cannot explain persistence of high mortality in early larval instars. Nowadays, extremely high temperatures typically occur only for a few days per year in central Europe. Long oviposition period (ca. 3 months) and high fecundity (ca. 1500 eggs) in *H. axyridis* may thus overcome low tolerance for high temperatures at the early stages, as only a small fraction of offspring is affected.

Interestingly, most traits of individuals that survived the exposure to elevated temperatures during the egg and early larval stages did not differ from control beetles reared at a constant 20°C (see Table S2, Figures S1 and S2). This indicates either that surviving individuals were not seriously affected by the elevated temperature experienced during early development, or alternatively that adult phenotype was corrected during following developmental stages (i.e. later larval instars and pupa). Correction of phenotype during late larval stages has been shown for the hawkmoth *Manduca sexta* [56]. Egg temperature affected larval growth in early instars in *M. sexta*, however adults traits (e.g. mass and fecundity) were not affected. In insects, larval growth is affected mainly by temperature and food availability. *H. axyridis* can compensate for food stress experienced in the early larval stages by increased growth in the later developmental stages [57]. However, food stress experience in the later larval instars can have a substantial effect on adult body size [58]. Our results indicate that the same is true for the effects of elevated temperature.

There is an intriguing contrast in the effects of elevated temperature on dry mass and structural body size. Periods of elevated temperature caused an increase in dry mass, especially when applied during late developmental stages. This contradicts the temperature-size rule and opposes the pattern reported for emergence of the temperature-size rule throughout the ontogeny in crustaceans [6]. Previous study confirm that Central European populations of *H. axyridis* seem to brake the temperature-size relationship as beetles reared at 30°C tend to have slightly higher body mass than beetles reared at 20°C [59]. In contrast, structural body size significantly decreased when *H. axyridis* was exposed to elevated temperatures during the last larval instar. The inconsistency between these two measures of body size has been reported before, in studies on sexual size dimorphism in insects (e.g., [60]). It was explained by sex-specific optimal body mass and body shape.

Strobbe and Stoks [61] also pointed out that the difference between reaction norms for body mass and structural size could be caused by the disparate nature of these two traits. Whilst structural size is fixed at adult emergence, body mass can change during the lifetime of the adult. However, the exact mechanisms causing this disparate effect of elevated temperature on body mass and structural body size in *H. axyridis* are unknown. We suggest that they would provide an interesting topic for future research. Nevertheless, some evidence exists that insects reared at higher temperatures have increased relative fat and protein content [62] and that the temperature experienced during the pupal stage has more serious effects on adult body mass than the temperature experienced in larval stages [63].

In agreement with Michie et al. [13], our data suggest that the critical period where temperature affects the degree of adult melanisation in *H. axyridis* is long. This should prevent unwanted effects of short fluctuations of ambient temperature. In contrast to Michie et al. [13] our results indicate that temperature influences the degree of adult melanisation more strongly during the later developmental stages. This is in agreement with the prediction of the theory of thermal melanism that sensitivity to environmental cues capable of determining adult colouration is expected to be as late as possible in preimaginal development [10], [12]. However, males of *H. axyridis* were less melanised than females, and this difference was exaggerated when high temperature exposure occurred during the pupal stage. Sexual dimorphism in colouration could be a result of sex-specific selection on viability [64], an interaction between sex-specific behaviour and colouration [65], [66] or due to mating preferences [67]. Moreover, there is a relationship between immune response, chemical defence and cuticular melanisation in insects, whereas sexual dimorphism in immune system and chemical defence exist [68]–[73]. Investment in immune system and chemical defence could be costly and thus vary in dependence on environmental conditions (e.g. temperature or diet during larval development) and these plastic responses can be sex-specific. Moreover, relatively broad ability of bird predators to generalize aposematic colour patterns of toxic insect suggests weak selective pressure for an uniform colouration [74].

A sex-specific phenotypic response to the timing of exposure to elevated temperature was observed also for body mass. Exposure during later stages increased the dry mass in *H. axyridis* males more than in females. Sex-specific differences in phenotypic plasticity due to rearing temperature have been previously identified in several insect species (e.g., [27], [75], [76]). However, information about the developmental stage at which these effects mainly act has been missing until now. To our knowledge, this is the first study to find a relationship between the developmental stage at which insects are exposed to elevated temperature and sex-specific adult phenotype.

Although several studies have been trying to explore effects of rearing temperature on particular developmental stages, experiments were based on comparison among individuals reared for their whole preimaginal development under particular constant or regularly fluctuating temperature treatments (e.g., [63], [77], [78]). Thus performance in a later developmental stage was affected by the temperature experienced before, which makes comparison of the temperature effects on later developmental stages problematical.

In conclusion, the timing of period at which elevated temperature was applied influenced juvenile survival rate, adult dry mass, structural body size and degree of melanisation in the harlequin ladybird, *Harmonia axyridis*. Moreover, the effects of this exposure on dry mass and melanisation rate were sex-specific, resulting in variation in sexual dimorphism caused by the timing of exposure period. Adult phenotype was affected most by elevated temperature experienced during the late developmental stages, especially the pupal stage. Early developmental stages suffered from increased mortality, but the adult phenotype of survivors was mostly unaffected by early exposure to elevated temperature. Detailed information on the effects of temperature on the biology of the highly invasive *H. axyridis* is crucial for predicting its invasion range and trends in its population growth. Therefore, future studies examining the effects of timing of period with elevated temperature during ontogeny on performance of *H. axyridis*, e.g. fecundity, mating success, longevity, desiccation rate, cold hardiness and immune function, are needed.

## Supporting Information

Figure S1 **The effect of period with elevated temperature during egg stage on survival rate.**
(DOC)

Figure S2 **The effect of period with elevated temperature during egg stage on adult body size and melanisation.**
(DOC)

Table S1 **Mean duration of particular life stage in** ***Harmonia axyridis.***
(DOC)

Table S2 **Results of post hoc testing for differences in body size and melanisation between sexes, treatments (timing of period with elevated temperature) and sex×treatment interactions.**
(DOC)
