# Supplementary material for: Gender and Timing during Ontogeny Matter: Effects of a Temporary High Temperature on Survival, Body Size and Colouration in Harmonia axyridis
Source: PLoS One. 2013 Sep 25;8(9):e74984. doi: 10.1371/journal.pone.0074984 (PMC3783448; doi:10.1371/journal.pone.0074984)
Supplement: Figure S1 — The effect of period with elevated temperature during egg stage on survival rate. (DOC) [file pone.0074984.s001.doc]

**Figure S1**

**The effect of period with elevated temperature during egg stage on survival rate**


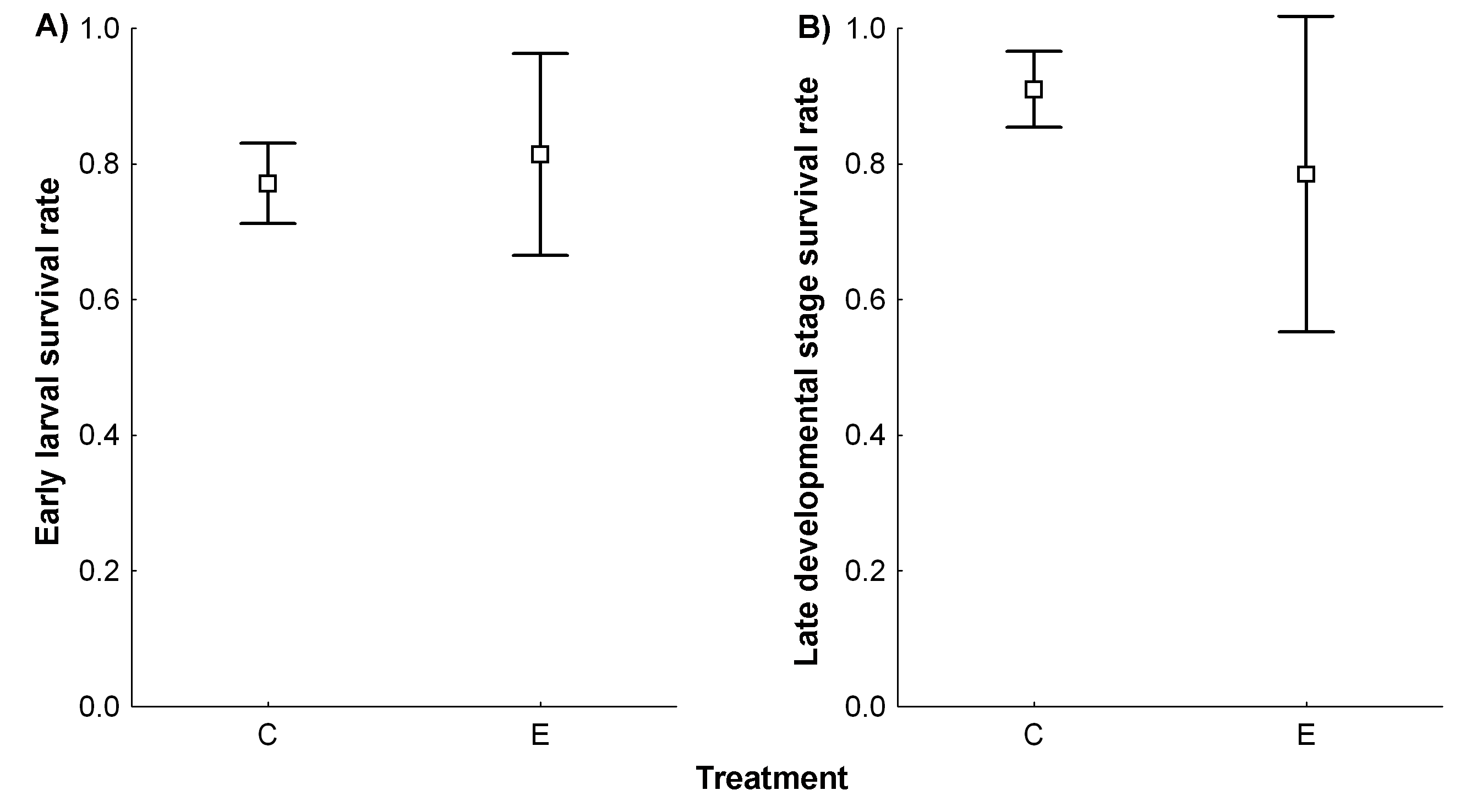


Survival rate of *Harmonia axyridis* was recorded for two stages. Early larval stage includes 1^st^ and 2^nd^ larval instars (panel **A**) whereas late developmental stage includes 3^rd^ and 4^th^ larval instars and pupa (panel **B**). Control individuals "C" were reared at constant temperature (20°C) and individuals from "E" treatment differed from control ones by exposition to a period of elevated temperature (48 hours at 33°C) during egg stage. Figure S1 and following analyses (see below) are based on limited dataset in comparison to results presented in result section of the paper, because for "E" treatment, 3^rd^ instar larvae were obtained only for 9 families (parental pairs). Mean and ± 1.96 SE are shown. There were no significant differences in early larval survival rate (GLMM-binomial: F_1,8_ = 0.26, P = 0.63) nor in late developmental stage survival rate (GLMM-binomial: F_1,8_ = 2.61, P = 0.15) between control individuals and those experienced period with elevated temperature during egg stage.
