# Supplementary material for: Gender and Timing during Ontogeny Matter: Effects of a Temporary High Temperature on Survival, Body Size and Colouration in Harmonia axyridis
Source: PLoS One. 2013 Sep 25;8(9):e74984. doi: 10.1371/journal.pone.0074984 (PMC3783448; doi:10.1371/journal.pone.0074984)
Supplement: Figure S2 — The effect of period with elevated temperature during egg stage on adult body size and melanisation. (DOC) [file pone.0074984.s002.doc]

**Figure S2**

**The effect of period with elevated temperature during egg stage on adult body size and melanisation**

**
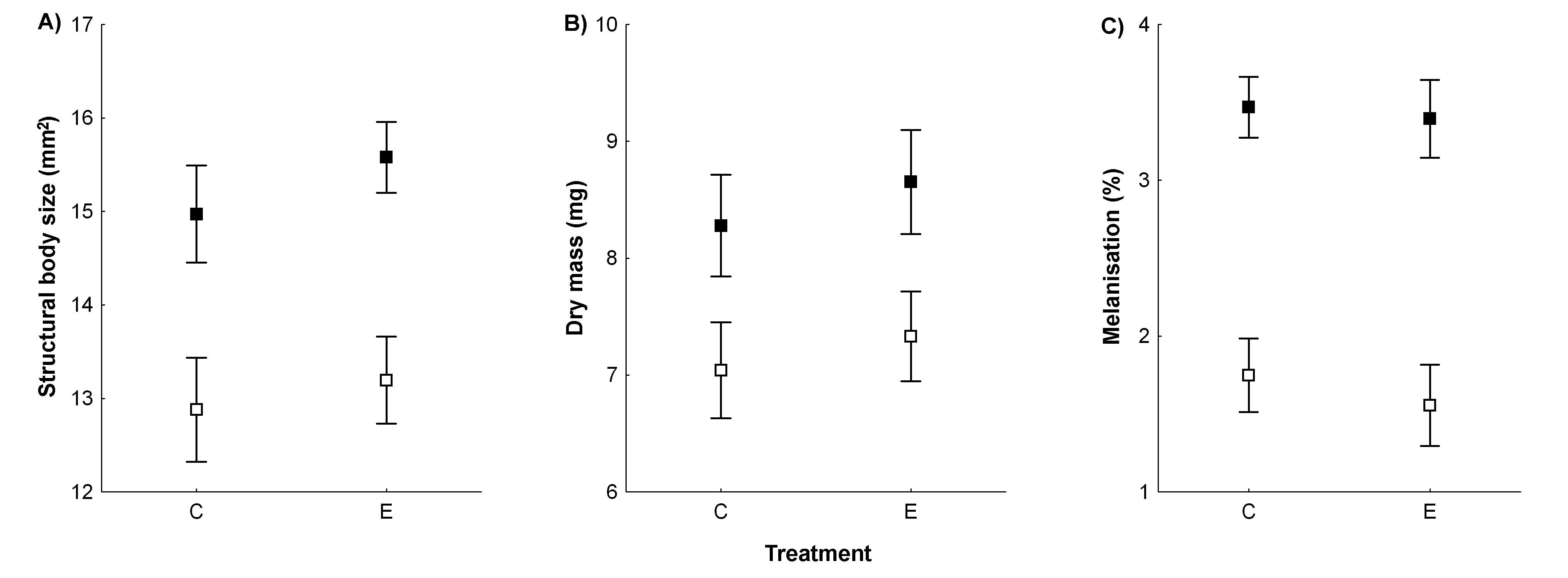
**

Control individuals "C" were reared at constant temperature (20°C) and individuals from "E" treatment differed from control ones by exposition to a period of elevated temperature (48 hours at 33°C) during egg stage. Mean and ± 1.96 SE are shown separately for males (open squares) and for females (close squares). Figure S2 and following analyses (see bellow) are based on limited dataset in comparison to results presented in result section of the paper, because for "E" treatment, adults of both sexes were obtained only for 6 families (parental pairs) from total of 12 included in the experiment. Structural body size (panel **A**) differed significantly between sexes (GLMM-gamma: F_1,83_ = 185.63; P < 0.001), was marginally affected by period with elevated temperature (GLMM-gamma: F_1,83_ = 4.05; P = 0.047) and was unaffected by interaction between sex and period with elevated temperature (GLMM-gamma: F_1,83_ = 0.01; P = 0.93). Dry mass (panel **B**) differed significantly between sexes (GLMM-gamma: F_1,83_ = 47.40; P < 0.001), but was unaffected by period with elevated temperature (GLMM-gamma: F_1,83_ = 0.61; P = 0.44) nor by interaction between sex and period with elevated temperature (GLMM-gamma: F_1,83_ = 0.07; P = 0.79). Melanisation of individuals (panel **C**) differed between sexes (GLMM-gamma: F_1,83_ = 241.06; P < 0.001), but was unaffected by period with elevated temperature (GLMM-gamma: F_1,83_ = 0.07; P = 0.80) nor by interaction between sex and period with elevated temperature (GLMM-gamma: F_1,83_ = 0.60; P = 0.44).
