# Supplementary material for: Gender and Timing during Ontogeny Matter: Effects of a Temporary High Temperature on Survival, Body Size and Colouration in Harmonia axyridis
Source: PLoS One. 2013 Sep 25;8(9):e74984. doi: 10.1371/journal.pone.0074984 (PMC3783448; doi:10.1371/journal.pone.0074984)
Supplement: Table S1 — Mean duration of particular life stage in Harmonia axyridis. (DOC) [file pone.0074984.s003.doc]

**Table S1**

**Mean duration of particular life stage in *Harmonia axyridis*.**

Beetles were reared at constant temperatures 20°C or 33°C, photoperiod 18L:6D and were supplied with food (pea aphids) and water ad libitum.

|  | Duration in days | |
| --- | --- | --- |
|  | 20°C | 33°C |
| Egg | 4.26 | 2.00^†^ |
| Larva 1-3 | 11.77 | 6.50^#^ |
| Larva 4 | 9.39 | 4.50 |
| Pupa | 7.39 | 3.62 |

^†^ Eggs suffered extremely high mortality at 33°C.

^#^ Approximate duration of particular larval instars at 33°C were: L1 - 1.0 day; L2 - 2.0 days; L3 - 3.5 days.
