# Supplementary material for: Gender and Timing during Ontogeny Matter: Effects of a Temporary High Temperature on Survival, Body Size and Colouration in Harmonia axyridis
Source: PLoS One. 2013 Sep 25;8(9):e74984. doi: 10.1371/journal.pone.0074984 (PMC3783448; doi:10.1371/journal.pone.0074984)
Supplement: Table S2 — Results of post hoc testing for differences in body size and melanisation between sexes, treatments (timing of period with elevated temperature) and sex×treatment interactions. (DOC) [file pone.0074984.s004.doc]

**Table S2**

**Results of post hoc testing for differences in body size and melanisation between sexes, treatments (timing of period with elevated temperature) and sex×treatment interactions.** For detailed description see text below.

| **Hypothesis tested** | **Dry mass** | | | | | **Structural body size** | | | | | **Melanisation** | | | | |
| --- | --- | --- | --- | --- | --- | --- | --- | --- | --- | --- | --- | --- | --- | --- | --- |
|  | **Estimate** | **Std. Error** | **z-value** | **P-value** | **P_adj_-value** | **Estimate** | **Std. Error** | **z-value** | **P-value** | **P_adj_-value** | **Estimate** | **Std. Error** | **z-value** | **P-value** | **P_adj_-value** |
| **C: M vs F == 0** | 0.02093 | 0.00324 | 6.466 | **<0.001** | **<0.001** | 0.01140 | 0.00098 | 11.579 | **<0.001** | **<0.001** | -0.66755 | 0.08021 | -8.322 | **<0.001** | **<0.001** |
| **L1: M vs F == 0** | 0.02059 | 0.00296 | 6.951 | **<0.001** | **<0.001** | 0.01199 | 0.00091 | 13.243 | **<0.001** | **<0.001** | -0.70015 | 0.07337 | -9.542 | **<0.001** | **<0.001** |
| **L3: M vs F == 0** | 0.01690 | 0.00298 | 5.669 | **<0.001** | **<0.001** | 0.01207 | 0.00097 | 12.426 | **<0.001** | **<0.001** | -0.60660 | 0.07656 | -7.923 | **<0.001** | **<0.001** |
| **L4: M vs F == 0** | 0.01117 | 0.00278 | 4.025 | **<0.001** | **<0.001** | 0.01013 | 0.00095 | 10.618 | **<0.001** | **<0.001** | -0.91181 | 0.08571 | -10.638 | **<0.001** | **<0.001** |
| **P: M vs F == 0** | 0.01033 | 0.00256 | 4.028 | **<0.001** | **<0.001** | 0.00988 | 0.00086 | 11.438 | **<0.001** | **<0.001** | -2.21417 | 0.15715 | -14.089 | **<0.001** | **<0.001** |
| **L1 vs C == 0** | -0.00187 | 0.00250 | -0.746 | 0.228 | 1.000 | -0.00099 | 0.00077 | -1.292 | 0.098 | 0.982 | -0.00906 | 0.05619 | -0.161 | 0.436 | 1.000 |
| **L3 vs C == 0** | -0.00242 | 0.00262 | -0.925 | 0.177 | 1.000 | 0.00176 | 0.00082 | 2.146 | **0.016** | 0.159 | -0.08840 | 0.05922 | -1.493 | 0.068 | 0.677 |
| **L4 vs C == 0** | -0.00553 | 0.00251 | -2.201 | **0.014** | 0.139 | 0.00277 | 0.00080 | 3.451 | **<0.001** | **0.003** | -0.15202 | 0.05884 | -2.584 | **0.005** | **0.049** |
| **P vs C == 0** | -0.00661 | 0.00250 | -2.644 | **0.004** | **0.041** | 0.00058 | 0.00078 | 0.737 | 0.231 | 1.000 | -0.42264 | 0.06180 | -6.839 | **<0.001** | **<0.001** |
| **L3 vs L1 == 0** | -0.00055 | 0.00245 | -0.226 | 0.411 | 1.000 | 0.00274 | 0.00077 | 3.558 | **0.000** | **0.002** | -0.07934 | 0.05633 | -1.409 | 0.079 | 0.794 |
| **L4 vs L1 == 0** | -0.00367 | 0.00234 | -1.568 | 0.058 | 0.584 | 0.00376 | 0.00075 | 4.980 | **<0.001** | **<0.001** | -0.14296 | 0.05598 | -2.554 | **0.005** | 0.053 |
| **P vs L1 == 0** | -0.00474 | 0.00233 | -2.038 | **0.021** | 0.208 | 0.00157 | 0.00074 | 2.129 | **0.017** | 0.166 | -0.41358 | 0.05906 | -7.003 | **<0.001** | **<0.001** |
| **L4 vs L3 == 0** | -0.00311 | 0.00246 | -1.267 | 0.103 | 1.026 | 0.00101 | 0.00081 | 1.258 | 0.104 | 1.000 | -0.06362 | 0.05843 | -1.089 | 0.138 | 1.000 |
| **P vs L3 == 0** | -0.00419 | 0.00244 | -1.717 | **0.043** | 0.430 | -0.00118 | 0.00079 | -1.498 | 0.067 | 0.671 | -0.33424 | 0.06158 | -5.428 | **<0.001** | **<0.001** |
| **P vs L4 == 0** | -0.00107 | 0.00235 | -0.458 | 0.323 | 1.000 | -0.00219 | 0.00078 | -2.825 | **0.002** | **0.024** | -0.27062 | 0.06155 | -4.396 | **<0.001** | **<0.001** |
| **C vs L1, M vs F == 0** | -0.00034 | 0.00440 | -0.078 | 0.469 | 1.000 | 0.00059 | 0.00134 | 0.438 | 0.331 | 1.000 | -0.03260 | 0.10900 | -0.299 | 0.382 | 1.000 |
| **C vs L3, M vs F == 0** | -0.00403 | 0.00439 | -0.919 | 0.179 | 1.790 | 0.00068 | 0.00138 | 0.490 | 0.312 | 1.000 | 0.06095 | 0.11063 | 0.551 | 0.291 | 1.000 |
| **C vs L4, M vs F == 0** | -0.00976 | 0.00426 | -2.290 | **0.011** | 0.110 | -0.00127 | 0.00137 | -0.926 | 0.177 | 1.000 | -0.24425 | 0.11729 | -2.082 | **0.019** | 0.187 |
| **C vs P, M vs F == 0** | -0.01061 | 0.00412 | -2.575 | **0.005** | **0.050** | -0.00152 | 0.00131 | -1.163 | 0.122 | 1.000 | -1.54661 | 0.17636 | -8.770 | **<0.001** | **<0.001** |
| **L1 vs L3, M vs F == 0** | -0.00369 | 0.00421 | -0.877 | 0.190 | 1.000 | 0.00009 | 0.00133 | 0.066 | 0.474 | 1.000 | 0.09355 | 0.10618 | 0.881 | 0.189 | 1.000 |
| **L1 vs L4, M vs F == 0** | -0.00942 | 0.00405 | -2.327 | **0.010** | 0.100 | -0.00186 | 0.00131 | -1.416 | 0.078 | 0.784 | -0.21166 | 0.11272 | -1.878 | **0.030** | 0.302 |
| **L1 vs P, M vs F == 0** | -0.01026 | 0.00393 | -2.609 | **0.005** | **0.045** | -0.00211 | 0.00126 | -1.677 | **0.047** | 0.468 | -1.51402 | 0.17354 | -8.724 | **<0.001** | **<0.001** |
| **L3 vs L4, M vs F == 0** | -0.00572 | 0.00407 | -1.407 | 0.080 | 0.797 | -0.00195 | 0.00136 | -1.430 | 0.076 | 0.764 | -0.30521 | 0.11472 | -2.661 | **0.004** | **0.039** |
| **L3 vs P, M vs F == 0** | -0.00657 | 0.00392 | -1.675 | **0.047** | 0.470 | -0.00220 | 0.00130 | -1.691 | **0.045** | 0.454 | -1.60756 | 0.17481 | -9.196 | **<0.001** | **<0.001** |
| **L4 vs P, M vs F == 0** | -0.00085 | 0.00380 | -0.223 | 0.412 | 1.000 | -0.00025 | 0.00130 | -0.193 | 0.423 | 1.000 | -1.30236 | 0.17938 | -7.260 | **<0.001** | **<0.001** |

Presented post hoc tests are based on GLMMs reported in main text of the paper. The models analyse effects of timing of period with elevated temperature on body size (structural body size or dry mass) and melanisation in adult beetles. Particular treatments employed were:
"C" - beetles reared at constant temperature (20°C); "L1" - beetles reared at constant temperature (20°C) with the exception of period with elevated temperature (48 hours at 33°C) during 1^st^ and partially 2^nd^ larval instar; "L3" - beetles reared at constant temperature (20°C) with the exception of period with elevated temperature (48 hours at 33°C) during 3^rd^ larval instar; "L4" - beetles reared at constant temperature (20°C) with the exception of period with elevated temperature (48 hours at 33°C) during 4^th^ larval instar; "P" - beetles reared at constant temperature (20°C) with the exception of period with elevated temperature (48 hours at 33°C) during pupa. Estimates, standard errors and z-values reported were obtained using glht function implemented in R package "multcomp" (Hothorn et al. 2008; R Development Core Team 2010). Reported P-values were computed directly from z-values. Adjusted probabilities (P_adj_-value) were adjusted using bonferroni correction to make the significance of partial contrast really conservative. Bonferroni correction was applied within a particular term, so: 1) in assessing significance of sexual dimorphism (difference between sexes within treatments; M = males; F = females), there were 5 contrasts (upper part of the table); 2) in assessing partial differences between treatments, there were 10 contrasts (middle part of the table); 3) in assessing partial differences in sex×treatment interactions, there were 10 contrasts (lower part of the table). All significant differences (P_adj_-value < 0.05) are highlighted in **bold**.

**References:**

Hothorn T, Bretz F and Westfall P (2008) Simultaneous Inference in General Parametric Models. *Biometrical Journal* 50: 346-363.

R Development Core Team (2010) A language and environment for statistical computing. Vienna. ISBN 3-900051-07-0, URL: [http://www.R-project.org](http://www.R-project.org/).
